# Associação entre as condições de nascimento e a densidade mineral óssea de adultos das coortes de nascimentos de 1982 e 1993 de Pelotas, Rio Grande do Sul, Brasil 

**DOI:** 10.1590/0102-311XPT085523

**Published:** 2024-03-11

**Authors:** Luísa Silveira da Silva, Ana Maria Baptista Menezes, Fernando C. Barros, Fernando C. Wehrmeister, Helen Denise Gonçalves da Silva, Bernardo Lessa Horta

**Affiliations:** 1 Universidade Federal de Pelotas, Pelotas, Brasil.; 2 Universidade Católica de Pelotas, Pelotas, Brasil.

**Keywords:** Bone Density, Birth Weight, Gestational Age, Fetal Growth Retardation, Densidade Óssea, Peso ao Nascer, Idade Gestacional, Retardo do Crescimento Fetal, Densidad Ósea, Peso al Nacer, Edad Gestacional, Retardo del Crescimiento Fetal

## Abstract

Este estudo avaliou a associação do peso ao nascer, idade gestacional e crescimento intrauterino com a densidade mineral óssea (DMO) aos 22 e 30 anos, nas coortes de nascimentos de 1982 e 1993 de Pelotas, Rio Grande do Sul, Brasil. A DMO foi medida por absorciometria por raios X com dupla energia (DXA), a associação foi avaliada usando análise de variância e a regressão linear múltipla para o controle de confundimento por: sexo, renda familiar ao nascer, tabagismo materno na gestação, escolaridade materna, cor da pele materna e índice de massa corporal pré-gestacional. Foi testado se a gordura corporal na vida adulta era mediadora da associação analisada, por meio da *G-computation Formula*. Foram avaliados 6.803 participantes das coortes de 1982 e 1993, aos 30 e 22 anos, respectivamente. O peso ao nascer teve associação com a DMO em todos os sítios, com maior diferença no colo femoral. Os nascidos com menos de 2.000g apresentaram, em média, -0,036g/cm^2^ (IC95%: -0,064; -0,008) de DMO no colo femoral em comparação àqueles com mais de 3.500g. Aqueles com escore-z de crescimento intrauterino com pelo menos 1,28 desvio padrão abaixo da média apresentaram, em média, -0,013g/cm^2^ (IC95%: -0,024; -0,002) de DMO na coluna lombar, em relação aos com escore-z acima da média. A análise de mediação mostrou que gordura corporal na idade adulta não mediou a associação. As condições de nascimento foram associadas com a densidade mineral óssea na vida adulta, e a identificação dos fatores precoces relacionados à perda de DMO é essencial devido à inversão demográfica em progresso em países de média e baixa renda.

## Abstract (en)

This study assessed the association of birth weight, gestational age, and intrauterine growth with bone mineral density (BMD) at 22 and 30 years of age in the 1982 and 1993 birth cohorts in Pelotas, Rio Grande do Sul State, Brazil. BMD was measured by dual-energy X-ray absorptiometry (DXA) and the association was assessed using analysis of variance. Multiple linear regression was used to control for confounding factors: sex; household income at birth; maternal smoking during pregnancy; maternal schooling; maternal ethnicity/skin color; and pre-pregnancy body mass index. The study tested whether body fat in adulthood was a mediator of the association analyzed, using the G-computation Formula. A total of 6,803 participants from the 1982 and 1993 cohorts were evaluated at 30 and 22 years of age, respectively. Birth weight was associated with BMD at all sites, with a greater difference at the femoral neck. Individuals born weighing less than 2,000g had on average -0.036g/cm^2^ (95%CI: -0.064; -0.008) of BMD in the femoral neck than individuals weighing more than 3,500g. Individuals with an intrauterine growth z-score at least 1.28 standard deviation below the mean had an average of -0.013g/cm^2^ (95%CI: -0.024; -0.002) of BMD in the lumbar spine compared with individuals with an above-average z-score. The mediation analysis showed that body fat in adulthood did not mediate the association. Birth conditions have been associated with BMD in adulthood and the identification of early factors related to bone loss is essential due to the demographic inversion that has been taking place in low- and middle-income countries.

## Resumen

Este estudio evaluó la asociación del peso al nacer, la edad gestacional y el crecimiento intrauterino con la densidad mineral ósea (DMO) a los 22 y 30 años de edad, en las Cohortes de Nacimiento de 1982 y 1993 de Pelotas, Rio Grande do Sul, Brasil. La DMO se midió mediante absorciometría de rayos X de doble emisión (DXA), y la asociación se evaluó mediante ANOVA y regresión lineal múltiple para controlar la confusión por sexo, ingresos familiares al nacer, tabaquismo materno durante el embarazo, escolaridad materna, color de piel materno e índice de masa corporal antes del embarazo. Se comprobó si la grasa corporal en la edad adulta era un mediador de la asociación analizada, utilizando *G-computation Formula*. Se evaluaron 6.803 participantes de las cohortes 82 y 93, de 30 y 22 años, respectivamente. El peso al nacer se asoció con la DMO en todos los sitios, con la mayor diferencia en el cuello femoral. Los nacidos con un peso inferior a 2.000g tuvieron una media de -0,036g/cm^2^ (IC95%: -0,064; -0,008) de DMO en el cuello femoral, que aquellos con más de 3.500g. Aquellos con una puntuación z de crecimiento intrauterino de al menos 1,28 desviaciones estándar por debajo de la media presentaron un promedio de -0,013g/cm^2^ (IC95%: -0,024; -0,002) de DMO en la columna lumbar, con relación a aquellos con un puntaje z superior a la media. El análisis de mediación mostró que la grasa corporal en la edad adulta no medió la asociación. Las condiciones de nacimiento se asociaron con la DMO en la edad adulta, y la identificación temprana de factores relacionados con la pérdida de DMO es esencial debido a la inversión demográfica que ha estado ocurriendo en los países de ingresos medios y bajos.

## Introdução

A densidade mineral óssea (DMO) é uma medida da quantidade de minerais, que compõem a microarquitetura óssea, como cálcio e fósforo, nos ossos. A diminuição da DMO está relacionada ao desenvolvimento da osteoporose, que, principalmente em idades mais avançadas, aumenta o risco de fraturas ^1^. De acordo com a Fundação Internacional de Osteoporose (International Osteoporosis Foundation, IOF), após os 60 anos, uma em cada três mulheres e um em cada cinco homens sofrem fraturas devido à fragilidade óssea ^2^.

A DMO aumenta entre o nascimento e o início da idade adulta, atingindo o seu pico por volta dos 25 anos de idade, tendendo a estabilizar-se até os 45-50 anos, quando começa a ocorrer um declínio gradual. Nas mulheres, ocorre uma aceleração do declínio após o início da menopausa ^1^. Nos Estados Unidos, a prevalência de osteoporose entre mulheres de 50-59 anos de idade é de 15,4%, aumentando para 34,9% naquelas com mais de 80 anos; já entre os homens, a prevalência é ao redor de 11% mesmo após os 80 anos de idade ^3^. De acordo com a Associação Brasileira de Avaliação Óssea e Osteometabolismo (Abrasso), aproximadamente 10 milhões de pessoas têm osteoporose no Brasil; no entanto, muitas pessoas não têm conhecimento da doença. Assim, apenas 20% dos indivíduos com a doença sabem de seu diagnóstico ^2^.

Evidências indicam que fatores precoces estariam associados à DMO e vários estudos têm relatado que o peso ao nascer e a idade gestacional estão diretamente relacionados a ela, tanto na infância como na vida adulta ^4^^,^^5^^,^^6^. No tocante ao crescimento intrauterino, menor número de estudos avaliou essa associação e os resultados são heterogêneos. Laitinen et al. ^7^ observaram maior prevalência de baixa DMO entre indivíduos que sofreram retardo de crescimento intrauterino; já Smith et al. ^8^ relataram que o retardo de crescimento estava associado com maior perda de DMO na idade adulta, enquanto Hovi et al. ^9^ não observou tal associação. Além das condições de nascimento, a dieta materna com a ingestão de minerais, como o magnésio, fósforo e potássio, e com baixo teor de gordura durante a gestação estaria relacionada positivamente à DMO dos filhos ^10^. Ainda, no que diz respeito a fatores associados à DMO, destaca-se a obesidade, cujo efeito no metabolismo ósseo foi abordado por alguns estudos. Por meio de marcadores bioquímicos, mecanismos físicos e biológicos, de alguma forma, a obesidade impactaria positivamente na DMO ^11^^,^^12^^,^^13^.

Tendo em vista a importância de identificar fatores precoces que estejam associados à DMO, bem como o pequeno número de estudos que avaliaram sua associação com o crescimento intrauterino, especialmente na vida adulta, decidimos conduzir este estudo para avaliar a relação do peso ao nascer, da idade gestacional e do crescimento intrauterino com a DMO (corpo inteiro, colo femoral e coluna lombar), além de analisar se essa associação é mediada pela gordura corporal em adultos participantes das coortes de nascimentos de 1982 e 1993 de Pelotas, Rio Grande do Sul, Brasil.

## Métodos

Foram utilizados dados de duas coortes de nascimentos realizadas na cidade de Pelotas. Nos anos de 1982 e 1993, todas as maternidades da cidade foram visitadas diariamente, os nascimentos identificados e os recém-nascidos cuja família residia na zona urbana da cidade foram examinados e as mães entrevistadas, logo após o nascimento. Esses indivíduos foram acompanhados prospectivamente em diferentes idades ^14^^,^^15^.

Entre os meses de junho de 2012 e fevereiro de 2013, tentou-se acompanhar todos participantes da coorte de 1982, sendo que 3.701 indivíduos foram avaliados, com uma idade média de 30,2 anos, que, adicionados aos 325 óbitos identificados entre os participantes das coortes, representaram uma taxa de acompanhamento de 68,1% ^15^. Na coorte de 1993, entre outubro de 2015 e julho de 2016, foram acompanhados 3.810 indivíduos de um total de 5.249, com idade média de 22,6 anos, que, junto aos 193 óbitos, representaram uma taxa de acompanhamento de 76,3% ^14^.

O peso ao nascer foi medido logo após o parto pelos funcionários dos hospitais, usando balanças pediátricas, calibradas semanalmente pela equipe de pesquisa, e a informação sobre essa variável foi coletada mediante consulta aos registros hospitalares. O peso inferior a 2.500g foi usado como ponto de corte para definir a ocorrência de baixo peso ao nascer ^16^. A idade gestacional foi calculada a partir do relato da mãe sobre a data da última menstruação, e caso a mãe não soubesse ou não se lembrasse, esse dado era considerado como *missing*. Foram considerados prematuros os indivíduos nascidos com menos de 37 semanas de idade gestacional ^17^. Para avaliar o crescimento intrauterino, foi estimado o escore-z do peso ao nascer, da idade gestacional e do sexo, usando como referência a população do Projeto INTERGROWTH-21st, o escore-z < -1,28 desvio padrão foi usado como ponto de corte para definir a ocorrência de retardo de crescimento intrauterino ^18^.

Nos dois estudos, a DMO foi avaliada usando a absorciometria por raios X com dupla energia (DXA), modelo Lunar GE (https://www.gehealthcare.com.br/). A DMO, em gramas por centímetro quadrado (g/cm^2^), foi medida no corpo inteiro, na coluna lombar e no colo femoral. Foram excluídas as gestantes, mulheres com suspeita de gravidez, participantes com próteses metálicas fixas em qualquer parte do corpo, com peso corporal acima de 120kg ou que estivessem em tratamento com suplemento de cálcio.

A análise dos dados utilizou o pacote estatístico Stata versão 17.0 (https://www.stata.com). Inicialmente, as médias de DMO foram comparadas usando a análise de variância (Anova), e a regressão linear múltipla foi usada para o controle de confundimento pelos potenciais fatores: sexo (masculino; feminino); tabagismo materno na gestação (fumou; parou de fumar na gravidez; nunca fumou); renda familiar ao nascer em salários mínimos (≤ 1; 1,1 a 3; 3,1 a 6; 6,1 a 10; > 10); escolaridade materna no perinatal (anos completos de estudo); cor da pele materna (avaliada pelo entrevistador); e o índice de massa corporal (IMC) materno pré-gestacional. A escolha da utilização da Anova e da regressão linear foi baseada na avaliação da distribuição da variável de DMO, a partir da verificação de valores de *skewness* (próximo de 0) e *kurtosis* (próximo de 3), proximidade dos valores de média e mediana, além da inspeção visual por meio de histograma. A normalidade dos resíduos foi avaliada pelo teste de Shapiro-Wilk e a homoscedasticidade foi verificada a partir do erro quadrático médio de cada associação.

Em um primeiro momento, a análise foi estratificada por coorte, a fim de identificar se havia diferença na média de DMO entre as coortes de 1982 e 1993, visto que os participantes que nasceram em 1982, aos 30 anos (Material Suplementar - Tabela S1; Supplementary Materialhttps://cadernos.ensp.fiocruz.br/static//arquivo/1678-4464-csp-40-03-PT085523-s.pdf), tinham passado recentemente pelo pico de massa óssea, enquanto os participantes que nasceram em 1993, aos 22 anos, ainda não atingiram o pico (Material Suplementar - Tabela S2; Supplementary Materialhttps://cadernos.ensp.fiocruz.br/static//arquivo/1678-4464-csp-40-03-PT085523-s.pdf)). Contudo, como não foi observada diferença entre os estratos, as análises foram realizadas com as duas coortes agrupadas. O teste de Wald foi usado para avaliar a interação das variáveis de DMO com sexo e ano da coorte, adotando-se valor de p < 0,10. Em todas as análises, foi calculado o valor de p de tendência e categórico e o resultado com menor valor foi apresentado. Os testes de hipóteses utilizaram um nível de 5% de significância.

A fim de detectar a existência de possível confusão residual, o tabagismo na vida adulta foi utilizado como controle negativo do desfecho, medido nas visitas aos participantes de 22 e 30 anos das coortes de 1993 e 1982, respectivamente. Por meio de questionário, em ambos os casos, foram abordadas as seguintes questões: “Tu já tiveste o costume de fumar pelo menos uma vez por semana?” (não; sim) e “Tu ainda fumas?” (não; sim); a partir dessas duas questões, foi construída a variável de tabagismo na vida adulta (não; sim), sendo considerado como “sim” quando a resposta das duas questões fosse positiva. O controle negativo tem como objetivo reproduzir uma condição que não envolva o mecanismo causal hipotético, mas as mesmas fontes de viés da associação analisada originariamente. Não havendo associação com o desfecho negativo, sugere-se que as associações observadas não decorrem de confundimento residual. Nesta análise, foram utilizadas as mesmas variáveis de exposição e as estimativas foram ajustadas para os mesmos fatores de confusão das análises que avaliaram a associação das condições de nascimento com a DMO. Uma vez que o desfecho negativo era uma variável dicotômica, utilizou-se a regressão de Poisson com ajuste robusto da variância para estimar a razão de prevalência.

A *G-computation Formula*^19^ foi usada para verificar se a associação das condições de nascimento com a DMO era mediada pela porcentagem de gordura corporal aos 22 e 30 anos. Nessas análises, foram incluídas, como base *confounders*, as variáveis: sexo, tabagismo materno na gestação, renda familiar ao nascer, escolaridade materna no perinatal, cor da pele materna e IMC materno pré-gestacional. O consumo de alimentos ultraprocessados, avaliado pelo questionário de frequência alimentar, elaborado a partir da lista de alimentos incluída no instrumento, proposto por Sichieri & Everhart ^20^ Schneider et al. ^21^, e a prática de atividade física em minutos aos 22 e 30 anos, medida por acelerômetro do modelo *Gravity Estimator of Normal Everyday Activity* (GENEA), foram considerados como *post-confounders*.

Os dois projetos foram aprovados pelos Comitês de Ética em Pesquisa da Faculdade de Medicina da Universidade Federal de Pelotas (UFPEL), registrados pelo ofício nº 16/12 (coorte de 1982) e protocolo nº 1.250.366 (coorte de 1993). Para cada acompanhamento, os participantes receberam um Termo de Consentimento Livre e Esclarecido (TCLE), constando informações sobre o estudo e endereço de contato dos pesquisadores responsáveis. Nos estudos perinatais de ambas as coortes, não houve entrega de TCLE, porém os respectivos protocolos foram aprovados pelo Comitê de Ética em Pesquisa.

## Resultados

Neste estudo, 6.803 participantes das coortes de 1982 e 1993 foram incluídos, por terem informações para pelo menos uma das variáveis referentes às condições de nascimento e à DMO. A Tabela 1 mostra que a prevalência de baixo peso ao nascer foi de 8,7%, enquanto 6,9% eram prematuros. Em relação ao crescimento intrauterino, a prevalência de retardo de crescimento intrauterino foi de 11,6%. No que diz respeito às condições socioeconômicas e demográficas, 64,5% dos participantes nasceram em famílias com renda inferior a três salários mínimos, cerca de metade das mães tinham até cinco anos completos de escolaridade (47%), um terço das mães relataram que fumaram durante a gestação (33,6%), e a maioria foi considerada como tendo cor da pele branca (79,3%).

**Tabela 1:** Características dos participantes das coortes de nascimentos de 1982 e 1993 de Pelotas, Rio Grande do Sul, Brasil.

Características	n	%
Sexo		
Masculino	3.263	48,0
Feminino	3.540	52,0
Peso ao nascer (g)		
< 2.000	121	1,8
2.000-2.499	469	6,9
2.500-2.999	1.779	26,2
3.000-3.499	2.593	38,1
≥ 3.500	1.838	27,0
Idade gestacional (semanas)		
< 37	421	6,9
37-40	4.858	79,8
> 40	810	13,3
Peso ao nascer de acordo com a idade gestacional e sexo (escore-z)		
< -1,28	706	11,6
-1,28 a 0	2.769	45,5
> 0	2.611	42,9
Renda familiar ao nascer (salários mínimos)		
≤ 1	1.259	18,7
1,1-3,0	3.080	45,8
3,1-6,0	1.472	21,9
6,1-10,0	480	7,1
> 10,0	432	6,5
Tabagismo materno na gestação		
Não	4.516	66,4
Sim	2.287	33,6
Escolaridade materna (anos)		
0-5	3.191	47,0
6-9	2.160	31,8
10-12	873	12,8
≥ 13	569	8,4
Cor da pele		
Branca	5.393	79,3
Não branca	1.407	20,7
IMC pré-gestacional (kg/m^2^)		
< 18,5	515	8,3
18,5-24,9	4.287	68,9
25,0-29,9	1.128	18,2
≥ 30,0	289	4,6
	Média	DP
DMO (g/cm^2^)		
Corpo inteiro	1,21	0,10
Colo femoral	1,08	0,15
Coluna lombar	1,22	0,14

DMO: densidade mineral óssea; DP: desvio padrão; IMC: índice de massa corporal.

A Tabela 2 apresenta a associação entre as condições de nascimento e a DMO de corpo inteiro, colo femoral e coluna lombar na vida adulta. Em relação às médias brutas de DMO, foi verificada tendência linear das médias conforme as categorias de peso ao nascer em todos os sítios, variando de 1,18g/cm^2^ a 1,23g/cm^2^ no corpo inteiro e na coluna lombar. A média de DMO no colo femoral manteve-se em 1,07g/cm^2^ nas três categorias de idade gestacional e crescimento intrauterino, enquanto os outros sítios mostraram aumento das médias de acordo com o aumento da idade gestacional e escore-z do crescimento intrauterino.

**Tabela 2:** Média bruta e análise ajustada da associação entre as condições de nascimento e densidade mineral óssea (DMO) do corpo inteiro, colo femoral e coluna lombar na vida adulta dos participantes das coortes de nascimentos de 1982 e 1993 de Pelotas, Rio Grane do Sul, Brasil.

Variáveis	DMO corpo inteiro (g/cm^2^)		DMO colo femoral (g/cm^2^)		DMO coluna lombar (g/cm^2^)	
	Média (IC95%)	Ajustado β (IC95%) *	Média (IC95%)	Ajustado β (IC95%) *	Média (IC95%)	Ajustado β (IC95%) *
Peso ao nascer (g)		p < 0,001 **		p = 0,002 **		p < 0,001 **
< 2.000	1,18 (1,16; 1,20)	-0,023 (-0,040; -0,006)	1,02 (0,99; 1,06)	-0,036 (-0,064; -0,008)	1,18 (1,15; 1,20)	-0,034 (-0,061; -0,008)
2.000-2.499	1,19 (1,18; 1,19)	-0,021 (-0,031; -0,012)	1,05 (1,03; 1,06)	-0,019 (-0,034; -0,003)	1,20 (1,19; 1,21)	-0,020 (-0,035; -0,005)
2.500-2.999	1,20 (1,19; 1,20)	-0,014 (-0,020; -0,008)	1,06 (1,05;1,07)	-0,006 (-0,016; 0,003)	1,20 (1,20; 1,21)	-0,013 (-0,022;-0,003)
3.000-3.499	1,21 (1,21; 1,21)	-0,007 (-0,012; -0,001)	1,07 (1,07; 1,08)	0,001 (-0,008; 0,009)	1,22 (1,21; 1,22)	-0,002 (-0,011; 0,005)
≥ 3500	1,23 (1,22; 1,23)	Referência (0)	1,08 (1,08; 1,09)	Referência (0)	1,22 (1,22; 1,230)	Referência (0)
Idade gestacional (semanas)		p = 0,007 **		p = 0,504 **		p = 0,003 **
< 37	1,20 (1,19; 1,21)	-0,012 (-0,023;0,002)	1,07 (1,05; 1,08)	0,004 (0,013; 0,021)	1,20 (1,190; 1,210)	-0,023 (-0,040; 0,006)
37-40	1,21 (1,21; 1,21)	-0,009 (-0,015; -0,002)	1,07 (1,07;1,08)	0,005 (-0,005; 0,016)	1,21 (1,210; 1,220)	-0,013 (-0,024; 0,002)
> 40	1,22 (1,21; 1,22)	Referência (0)	1,07 (1,06; 1,08)	Referência (0)	1,23 (1,220; 1,240)	Referência (0)
Crescimento intrauterino (escore-z)		p < 0,001 **		p = 0,070 **		p = 0,007 **
< -1,28	1,20 (1,20; 1,21)	-0,011 (-0,019; -0,004)	1,07 (1,05; 1,08)	-0,013 (-0,025; -0,001)	1,20 (1,19; 1,21)	-0,015 (-0,027; 0,003)
-1,28 a 0,00	1,21 (1,20; 1,21)	-0,007 (-0,012; 0,002)	1,07 (1,07; 1,08)	-0,002 (-0,010; 0,005)	1,21 (1,21; 1,22)	-0,006 (-0,014; 0,001)
> 0,00	1,21 (1,21; 1,22)	Referência (0)	1,07 (1,07; 1,08)	Referência (0)	1,22 (1,21; 1,23)	Referência (0)

IC95%: intervalo de 95% de confiança; IMC: índice de massa corporal.

* Ajustado para: sexo, tabagismo materno na gestação, renda familiar ao nascer, escolaridade materna, cor da pele materna e IMC pré-gestacional;

** Valor de p de tendência linear.

O peso ao nascer mostrou associação positiva com a DMO em todos os sítios. A maior diferença observada no colo femoral foi entre os indivíduos que nasceram com peso menor de 2.000g, que apresentaram, em média, -0,036g/cm^2^ (IC95%: -0,064; -0,008) de DMO em relação aos que nasceram com mais de 3.500g. Mesmo após o ajuste para possíveis fatores de confusão, a idade gestacional apresentou associação positiva com a DMO do corpo inteiro (p = 0,007) e da coluna lombar (p = 0,003); indivíduos que nasceram antes de completar 37 semanas de gestação apresentaram, em média, -0,023g/cm^2^ (IC95%: -0,040; 0,006) de DMO da coluna lombar, quando comparados com os nascidos com mais de 40 semanas, enquanto para o colo femoral não foi observada associação. O crescimento intrauterino também apresentou associação positiva com a DMO do corpo inteiro e da coluna lombar; no colo femoral, após ajuste para fatores de confusão, foi observado que indivíduos com retardo de crescimento intrauterino tiveram, em média, -0,013g/cm^2^ (IC95%: -0,025; -0,001) de DMO comparado àqueles cujo peso ao nascer estava acima da média de acordo com a idade gestacional e sexo; entretanto, não houve diferença estatisticamente significativa. Nenhuma das associações foi modificada por sexo (p de interação > 0,15) (Tabela 2).

A Tabela 3 mostra que o tabagismo na vida adulta estava associado com o peso ao nascer na análise bruta, mas, após o ajuste para as variáveis de confusão, essa associação desapareceu. Em relação à idade gestacional e ao crescimento intrauterino, não foi observada associação com o tabagismo na vida adulta na análise bruta; no entanto, as razões de prevalência tiveram sua magnitude minimizada após o ajuste.

**Tabela 3:** Controle negativo da associação entre as condições de nascimento e tabagismo na vida adulta dos participantes das coortes de nascimentos de 1982 e 1993 de Pelotas, Rio Grande do Sul, Brasil.

Variáveis	Tabagismo	
	Análise bruta	Análise ajustada *
	RP (IC95%)	RP (IC95%)
Peso ao nascer (g)		
< 2.499	1,18 (1,01; 1,38)	1,13 (0,95; 1,34)
2.500-2.999	1,06 (0,94; 1,18)	1,03 (0,91; 1,16)
3.000-3.499	1,06 (0,95; 1,18)	1,06 (0,95; 1,19)
≥ 3.500	Referência (1,00)	Referência (1,00)
Idade gestacional (semanas)		
< 37	1,05 (0,88; 1,25)	1,01 (0,84; 1,21)
≥ 37	Referência (1,00)	Referência (1,00)
Crescimento intrauterino (escore-z)		
< -1,28	1,02 (0,89; 1,18)	0,94 (0,80; 1,09)
-1,28 a 0,00	1,05 (0,96; 1,16)	1,00 (0,90; 1,10)
> 0,00	Referência (1,00)	Referência (1,00)

IC95%: intervalo de 95% de confiança; RP: razão de prevalência.

* Análise ajustada para sexo, renda familiar ao nascer, fumo materno na gestação, escolaridade materna, cor da pele da mãe e índice de massa corporal pré-gestacional.

A análise de mediação mostrou que a porcentagem de gordura corporal na vida adulta capturou, no máximo, 33,3% da associação entre condições de nascimento e DMO nos sítios avaliados. Como o intervalo de confiança do efeito indireto incluiu a nulidade, ele não foi estatisticamente significativo. Dessa forma, não podemos descartar que a mediação observada pode ser estatisticamente igual a zero (Tabela 4).

**Tabela 4:** Análise de mediação da associação entre a porcentagem de gordura corporal e as condições de nascimento e a densidade mineral óssea (DMO) do corpo inteiro, colo femoral e coluna lombar da vida adulta dos participantes das coortes de nascimentos de 1982 e 1993 de Pelotas, Rio Grande do Sul, Brasil.

Variáveis	Efeito total β (IC95%)	Efeito natural indireto β (IC95%)	Mediado (%)
DMO corpo inteiro (g/cm^2^)			
Peso ao nascer (g)	0,008 (0,004; 0,013)	0,001 (-0,002; 0,004)	7,5
Idade gestacional (semanas)	0,006 (-0,001; 0,013)	0,002 (-0,002; 0,005)	33,3
Crescimento intrauterino (escore-z)	0,005 (-0,001; 0,010)	-0,001 (-0,004; 0,003)	0,0
DMO colo femoral (g/cm^2^)				
Peso ao nascer (g)	0,009 (0,002; 0,016)	0,001 (-0,004; 0,006)	11,1
Idade gestacional (semanas)	-0,004 (-0,015; 0,007)	0,001 (-0,005; 0,007)	0,0
Crescimento intrauterino (escore-z)	0,002 (-0,007; 0,011)	-0,001 (-0,008; 0,004)	0,0
DMO coluna lombar (g/cm^2^)				
Peso ao nascer (g)	0,010 (0,004; 0,017)	0,001 (-0,005; 0,006)	10,0
Idade gestacional (semanas)	0,010 (-0,001; 0,021)	0,001 (-0,005; 0,006)	10,0
Crescimento intrauterino (escore-z)	0,005 (-0,004; 0,013)	-0,001 (-0,007; 0,004)	0,0

IC95%: intervalo de 95% de confiança.

## Discussão

Este estudo mostrou que os indivíduos nascidos com baixo peso, prematuros e/ou com retardo de crescimento intrauterino apresentaram menor DMO no corpo inteiro e na coluna lombar na vida adulta (22 e 30 anos). Para o sítio colo femoral, a DMO também foi menor naqueles que nasceram com baixo peso ou com retardo de crescimento intrauterino. Essas associações não foram modificadas pelo sexo.

A relação do peso ao nascer com a DMO na vida adulta pode decorrer do processo de crescimento acelerado que é observado na primeira infância, visto que o peso ao nascer influencia o acúmulo de cálcio nos ossos de forma positiva ^5^^,^^22^. O peso ao nascer também está positivamente associado com os níveis basais do hormônio do crescimento e cortisol, que estão envolvidos tanto com o pico de massa óssea, que ocorre ao redor dos 25 anos de idade, quanto com sua perda na vida adulta. Portanto, o baixo peso ao nascer poderia influenciar no menor pico de massa óssea, assim como na perda acelerada da DMO durante a vida adulta ^23^. Duas revisões sistemáticas indicaram que a associação positiva entre peso ao nascer e saúde óssea na infância também é observada em adultos e adolescentes, mas com menor magnitude ^5^^,^^24^.

Cerca de 80% do acúmulo de minerais ósseos ocorre no terceiro trimestre da gestação; logo, um parto prematuro pode interromper o desenvolvimento ósseo fetal ^25^. Após o nascimento, é comum que ocorra queda dos níveis séricos de cálcio devido à interrupção do fornecimento de minerais e nutrientes pelo cordão umbilical; entretanto, geralmente há recuperação dos níveis ideais pelo aleitamento materno ^26^. Uma vez que recém-nascidos prematuros não têm absorção intestinal eficiente, essa recuperação por meio do aleitamento materno não ocorre de forma eficaz, causando deficiência desses minerais, que impactam diretamente na DMO após o nascimento, até a vida adulta, o que explicaria a associação negativa entre prematuridade e DMO na vida adulta ^25^.

Estudo de caso-controle realizado na Austrália apontou que, em relação aos adultos nascidos a termo, os prematuros apresentaram menor DMO, mas apenas quando a análise não foi ajustada para altura e peso ^6^. Em coorte de nascimentos de Montreal, Canadá, foi observado que os participantes que nasceram prematuros apresentaram menor DMO em relação aos que nasceram a termo, especialmente no colo femoral ^27^.

No nosso estudo, o retardo de crescimento intrauterino esteve associado com a menor DMO no corpo inteiro e na coluna lombar nas idades avaliadas. Uma possível razão para esse achado dá-se ainda na gestação. O transporte de cálcio e fosfato realizado pela placenta pode ser interrompido quando o feto está sofrendo restrição de crescimento, provocando osteopenia antes mesmo do nascimento ^28^. Sendo assim, o retardo de crescimento intrauterino pode ser resultante do tamanho insuficiente da placenta para atender às necessidades nutricionais do feto, principalmente no terceiro trimestre da gestação, promovendo alterações hormonais, para adaptar o organismo ao menor aporte calórico e proteico, e preservando órgãos vitais ^29^. As alterações hormonais advindas do retardo de crescimento na vida intrauterina podem, portanto, ser associadas negativamente aos níveis básicos de hormônio do crescimento (GH) na infância, pois esse hormônio ativa a formação e a reabsorção óssea por mecanismos diretos e indiretos, e, consequentemente, sua deficiência contribui para a diminuição na mineralização, gerando perda óssea na vida adulta ^29^^,^^30^^,^^31^.

Diferentemente deste estudo, Hovi et al. ^9^ não identificaram associação entre o retardo de crescimento intrauterino e a saúde óssea na vida adulta; no entanto, fizeram ajuste apenas para variáveis pós-concepção. Ao controlar a análise para variáveis pós-concepção, que são possíveis mediadores, caminhos causais podem ser bloqueados e a magnitude da associação entre exposição e desfecho possivelmente será subestimada. O estudo de caso-controle realizado com indivíduos de baixo peso e que sofreram retardo de crescimento intrauterino, tratados em unidade de terapia intensiva (UTI) neonatal, encontrou uma associação negativa com a perda de DMO na vida adulta ^8^. Embora nosso estudo tenha avaliado crianças que passaram por cuidados intensivos apenas na coorte de 1993, pois em 1982 ainda não havia UTI neonatal na cidade, os resultados apontam para a mesma direção. Esse desfecho também foi encontrado no estudo de Laitinen et al. ^7^, que analisaram a prevalência de baixa DMO e encontraram maiores prevalências entre quem sofrem retardo de crescimento intrauterino.

Entre as limitações deste estudo, está a forma como a idade gestacional foi avaliada. O relato materno da data da última menstruação pode estar sujeito a erro não diferencial, subestimando a magnitude da força de associação ^32^. Apesar disso, foi encontrada associação estatisticamente significativa; portanto, não podemos atribuir as associações observadas a um erro na medida da idade gestacional. Outra limitação relevante está no modelo de ajuste adotado, uma vez que a variável de dieta materna deveria ser incluída como fator de confusão; entretanto, essa informação não foi coletada no estudo.

Uma das qualidades deste estudo é o fato de ter medidas nas duas idades pelo DXA, um aparelho considerado padrão-ouro para a avaliação da massa óssea, com boa precisão e acurácia em suas medidas. Como demais vantagens, salienta-se o delineamento de coorte, tendo uma avaliação da massa óssea próximo do seu pico, possibilitando observar a relação das condições de nascimento com a DMO ao final do período de desenvolvimento ósseo. O uso de controle negativo também permitiu afirmar que as associações observadas não foram decorrentes de confusão residual, uma vez que a associação com o desfecho negativo não se mostrou estatisticamente significativa após o controle de confusão.

Apesar de não podermos inferir causalidade com base no delineamento utilizado, estudos realizados com crianças e adolescentes indicam que a magnitude da associação é minimizada na adolescência. Em adultos, a associação entre as condições de nascimento e a DMO têm mostrado a mesma direção; no entanto, existem poucos estudos avaliando adultos jovens, havendo necessidade de mais pesquisas com essa faixa etária. Considerando o fenômeno de inversão demográfica observado em países de média e baixa renda, a identificação de fatores precoces que estejam associados à perda de DMO torna-se essencial para a discussão, a investigação e o desenvolvimento de políticas de saúde pública voltadas à prevenção e manejo dos fatores de risco de doenças relacionadas à saúde óssea.
