# Supplementary material for: Association between birth conditions and bone mineral density in adults from the 1982 and 1993 birth cohorts in Pelotas, Rio Grande do Sul State, Brazil
Source: Cad Saude Publica. 2024 Mar 11;40(3):e00085523. [Article in Portuguese] doi: 10.1590/0102-311XPT085523 (PMC10929887; doi:10.1590/0102-311XPT085523)
Supplement: Supplementary file 1 [file 1678-4464-csp-40-03-PT085523-s.pdf]

Material suplementar

Tabela S1 Média bruta e análise ajustada da associação entre as condições de nascimento e densidade mineral óssea (DMO) do corpo inteiro, colo femoral e coluna lombar na vida adulta dos participantes da coorte de nascimentos de 1982 de Pelotas, Rio Grande do Sul, Brasil.

**Variáveis**	**DMO corpo inteiro (g/cm²)**		**DMO colo femoral (g/cm²)**		**DMO coluna lombar (g/cm²)**	
	**Média (IC95%)**	**Ajustado β (IC95%) ***	**Média (IC95%)**	**Ajustado β (IC95%) ***	**Média (IC95%)**	**Ajustado β (IC95%) ***
Peso ao nascer (g)		p < 0,001 *		p = 0,001 **		p < 0,001 **
< 2.000	1,18 (1,16; 1,20)	-0,034 (-0,058; -0,009)	0,98 (0,93;1,03)	-0,079 (-0,119; -0,040)	1,16 (1,13; 1,19)	-0,057 (-0,096; -0,017)
2.000-2.499	1,19 (1,18; 1,21)	-0,024 (-0,038; -0,010)	1,04 (1,02; 1,06)	-0,033 (-0,056; -0,010)	1,20 (1,18; 1,22)	-0,029 (-0,052; -0,005)
2500 a 2999	1,20 (1,19; 1,21)	-0,014 (-0,023; -0,006)	1,05 (1,04; 1,06)	-0,015 (-0,028; 0,001)	1,21 (1,20; 1,21)	-0,015 (-0,029; -0,001)
3.000-3.499	1,21 (1,21; 1,22)	-0,009 (-0,016; -0,001)	1,06 (1,05; 1,07)	-0,008 (-0,021; 0,003)	1,22 (1,21; 1,22)	-0,005 (-0,017; 0,007)
≥ 3.500	1,23 (1,23; 1,24)	Referência (0)	1,07 (1,06; 1,08)	Referência (0)	1,23 (1,22; 1,24)	Referência (0)
Idade gestacional (semanas)		p = 0,276 **		p = 0,334 **		p = 0,020 **
< 37	1,21 (1,19; 1,22)	-0,011 (-0,027; 0,004)	1,05 (1,02; 1,07)	0,014 (0,039; 0,011)	1,19 (1,17; 1,21)	-0,031 (-0,057; 0,006)
37-40	1,22 (1,21; 1,22)	-0,009 (-0,009; -0,006)	1,06 (1,05; 1,07)	0,003 (-0,015; 0,009)	1,22 (1,21; 1,22)	-0,009 (-0,021; 0,003)
> 40	1,21 (1,21; 1,22)	Referência (0)	1,07 (1,05; 1,07)	Referência (0)	1,23 (1,21; 1,23)	Referência (0)
Crescimento intrauterino (escore-z)		p < 0,002 **		p = 0,041 **		p = 0,005 **
< -1,28	1,21 (1,20; 1,22)	-0,016 (-0,026; -0,005)	1,05 (1,03; 1,06)	-0,018 (-0,035; -0,001)	1,20 (1,19; 1,21)	-0,025 (-0,042; -0,007)
-1,28 a 0,00	1,21 (1,20; 1,22)	-0,007 (-0,015; -0,001)	1,06 (1,05; 1,06)	-0,006 (-0,018; 0,005)	1,21 (1,21; 1,22)	-0,008 (-0,020; 0,003)
> 0,00	1,22 (1,22; 1,23)	Referência (0)	1,07 (1,06; 1,07)	Referência (0)	1,22 (1,21; 1,23)	Referência (0)

IC95%: intervalo de 95% de confiança.

* Ajustado para: sexo, tabagismo materno na gestação, renda familiar ao nascer, escolaridade materna, cor da pele materna e índice de massa corporal pré-gestacional.

** Valor de p de tendência linear;

Tabela S2 Média bruta e análise ajustada da associação entre as condições de nascimento e densidade mineral óssea (DMO) do corpo inteiro, colo femoral e coluna lombar na vida adulta dos participantes da coorte de nascimentos de 1993 de Pelotas, Rio Grande do Sul, Brasil.

**Variáveis**	**DMO corpo inteiro (g/cm²)**		**DMO colo femoral (g/cm²)**		**DMO coluna lombar (g/cm²)**	
	**Média (IC95%)**	**Ajustado β (IC95%) ***	**Média (IC95%)**	**Ajustado β (IC95%) ***	**Média (IC95%)**	**Ajustado β (IC95%) ***
Peso ao nascer (g)		p < 0,001 **		p = 0,301 **		p < 0,058 **
< 2.000	1,19 (1,16; 1,22)	-0,013 (-0,037; 0,010)	1,07 (1,03; 1,12)	0,003 (-0,036; 0,042)	1,19 (1,15; 1,23)	-0,015 (-0,051; 0,021)
2.000-2499	1,18 (1,17; 1,19)	-0,018 (-0,030; -0,005)	1,06 (1,04; 1,08)	-0,011 (-0,032;-0,009)	1,20 (1,18;1,22)	-0,012 (-0,031; 0,007)
2.500-2.999	1,19 (1,18; 1,20)	-0,014 (-0,022; -0,005)	1,07 (1,06; 1,08)	-0,001 (-0,014; 0,014)	1,20 (1,19; 1,21)	-0,010 (-0,023; 0,003)
3.000-3.499	1,21 (1,20; 1,21)	-0,005 (-0,013; 0,002)	1,09 (1,08; 1,10)	0,008 (-0,004; 0,021)	1,22 (1,21; 1,22)	-0,001 (-0,012; 0,011)
≥ 3.500	1,22 (1,21; 1,23)	Referência (0)	1,09 (1,08; 1,10)	Referência (0)	1,22 (1,21; 1,23)	Referência (0)
Idade gestacional (semanas)		p = 0,101 **		p = 0,130 **		p = 0,278 **
< 37	1,20 (1,18; 1,21)	-0,022 (-0,045; -0,001)	1,08 (1,06; 1,10)	-0,029 (-0,067; 0,007)	1,20 (1,19; 1,22)	-0,024 (-0,059; 0,010)
37-40	1,20 (1,20; 1,21)	-0,021 (-0,041; -0,001)	1,08 (1,08; 1,09)	-0,033 (-0,067; -0,001)	1,21 (1,21; 1,22)	-0,020 (-0,051; 0,010)
> 40	1,23 (1,21; 1,25)	Referência (0)	1,13 (1,10;1,16)	Referência (0)	1,24 (1,21; 1,27)	Referência (0)
Crescimento intrauterino (escore-z)		p < 0,017 **		p = 0,892 **		p = 0,186 **
< -1,28	1,20 (1,19; 1,21)	-0,010 (-0,021; 0,001)	1,09 (1,07;1,10)	-0,002 (-0,020; 0,016)	1,21 (1,19; 1,22)	-0,008 (-0,025; 0,008)
-1,28 a 0,00	1,20 (1,20; 1,21)	-0,007 (-0,013; -0,001)	1,09 (1,08; 1,09)	0,001 (-0,010; 0,011)	1,21 (1,20; 1,22)	-0,006 (-0,016; 0,003)
> 0,00	1,21 (1,20; 1,21)	Referência (0)	1,08 (1,07; 1,09)	Referência (0)	1,22 (1,21; 1,22)	Referência (0)

IC95%: intervalo de 95% de confiança.

** Ajustado para: sexo, tabagismo materno na gestação, renda familiar ao nascer, escolaridade materna, cor da pele materna e índice de massa corporal pré-gestacional;

** Valor de p de tendência linear.
